# Evaluation of a clinically efficient [^18^F]FDG PET/CT protocol: short dynamic and two-phase static imaging for differentiating malignant pulmonary lesions

**DOI:** 10.1186/s13550-026-01419-7

**Published:** 2026-04-02

**Authors:** Yingqin Jia, Rui Qiu, Chunlei Han, Fan Zhang, Sheng Zhang, Dan Zhao, Xiang-Guo Li, Xiaoqing Zhuang

**Affiliations:** 1 Nuclear Medicine Department, General Hospital of Ningxia Medical University, Yinchuan, China; 2 Department of General Thoracic Surgery, General Hospital of Ningxia Medical University, Yinchuan, China; 3 Turku PET Centre, University of Turku, Turku University Hospital, Turku, Finland; 4 Department of Radiology, Guangdong Provincial Hospital of Traditional Chinese Medicine, Zhuhai, China; 5 Department of Chemistry, University of Turku, Turku, Finland; 6 InFLAMES Research Flagship Center, University of Turku, Turku, Finland

**Keywords:** [^18^F]FDG PET/CT, Pulmonary lesions, Dynamic PET, Kinetic parameters, K_i_, Tumor-to-blood ratio, Delayed imaging, Diagnostic accuracy

## Abstract

**Background:**

Pulmonary lesions affect a large population globally, posing huge challenges in clinical management and public healthcare. Functional imaging-based diagnosis facilitates the classification and treatment planning for pulmonary lesions. [^18^F]FDG PET/CT is a valuable tool for evaluating pulmonary lesions, but it is limited by false-positive uptake in inflammatory processes. Although quantitative kinetic analysis can improve diagnostic specificity, long dynamic acquisitions pose practical challenges in clinical settings and compromise patient comfort. This study evaluated an efficient and clinically practical protocol combining a 10-min dynamic scan with two-phase static scanning to improve diagnostic accuracy.

**Results:**

Forty patients with pulmonary lesions confirmed by histopathology (28 malignant, 12 non-malignant) were included in the analysis. Kinetic parameters were derived from time-activity curves generated by integrating dynamic and static data points using different compartment models with image-derived input functions. Semi-quantitative parameters, including maximum standardized uptake value (SUV_max_), tumor-to-blood standard uptake ratio (SUR) from the early static scan, and delayed SUV_max_ and delayed SUR from the delayed static scan, were also calculated and assessed. Malignant lesions showed significantly higher net influx rates (K_i_) calculated from Patlak model compared with non-malignant lesions (*P* = 0.023). The receiver operating characteristic (ROC) curve analysis for K_i_ yielded an area under the curve (AUC) of 0.729. Both SUR from the standard static scan (*P* = 0.045, AUC = 0.702) and delayed SUR from the 2.5-h static scan (*P* = 0.029, AUC = 0.720) were significantly higher in malignant lesions and demonstrated significant diagnostic accuracy. Standard SUV_max_, and delayed SUV_max_ did not show significant differences between groups.

**Conclusions:**

The combined 10-min dynamic and two-phase static [^18^F]FDG PET/CT protocol may be feasible in selected centers with optimized scheduling. When applicable, this method allows derivation of the net influx rate K_i_ from Patlak graphical analysis as well as the easily calculated semi-quantitative parameters SUR and delayed SUR, all of which demonstrate significant diagnostic value for differentiating malignant from non-malignant pulmonary lesions, outperforming conventional SUV_max_.

## Background

Lung cancer is a significant public health issue worldwide, consistently ranking as the leading cause of cancer mortality in men and the second highest in women [1]. By 2050, the number of lung cancer cases is predicted to increase to 4.62 million new cases and 3.55 million deaths [2]. As the most effective approach to decrease the burden of lung cancer, the implementation of lung cancer screening has led to an increase in the diagnosis of early-stage lung cancer and a reduction in lung cancer mortality. Lung cancer screening with low-dose computed tomography (CT) is recommended for high-risk individuals [3, 4]. In clinical practice, it is essential to have sensitive and practical techniques for differentiating malignant pulmonary lesions from inflammatory and benign lesions. However, since CT is empirically based on the morphological features of pulmonary nodules, it is difficult to evaluate the malignancy of pulmonary lesions. Positron emission tomography (PET) with CT (PET/CT) imaging, by combining functional and anatomical information, offers high sensitivity and accuracy for evaluating most malignancies [5].

2-Deoxy-2-[^18^F]fluoroglucose ([^18^F]FDG) is a radiolabeled glucose analog widely used in PET/CT imaging. [^18^F]FDG is one of the PET radiopharmaceuticals that has the best global availability, which is a crucial factor for public healthcare sectors to implement nuclear imaging for broad clinical use. In general, malignant tumor cells exhibit enhanced glucose uptake and glycolysis, making [^18^F]FDG PET/CT a routine tool for diagnosing and monitoring treatment response for malignant tumors. In particular, it is one of the most common methods for evaluating pulmonary lesions. However, [^18^F]FDG uptake is not specific to malignancy, presenting an inherent limitation of false-positive findings commonly associated with infection or inflammation [6, 7]. Moreover, benign pulmonary lesions can exhibit mild to moderate [^18^F]FDG uptake, which complicates the characterization of malignant pulmonary lesions based solely on standard semi-quantitative metrics.

To overcome the limitations of [^18^F]FDG imaging and enhance diagnostic accuracy, several approaches have been developed to quantify [^18^F]FDG distribution over time. Dual time point static PET scanning, a semi-quantitative method involving two static PET examinations, has been implemented in clinical settings and has significantly improved assessment accuracy [8, 9]. A more quantitative approach involves full kinetic analysis derived from dynamic PET acquisitions and time-activity curves (TACs). However, the requirement for a 60-min PET dynamic acquisition poses significant practical challenges in clinical settings. The extended scan duration is often incompatible with patient comfort, particularly for individuals with respiratory conditions or limited mobility.

The goal of this study was to evaluate a more efficient and clinically practical [^18^F]FDG PET/CT protocol for characterizing pulmonary lesions. By significantly reducing the dynamic scan duration to 10 min and integrating it with a two-phase static scan, we aim to improve patient comfort, streamline the clinical workflow, and preserve diagnostic accuracy for malignant pulmonary lesions. Kinetic parameters were calculated by combining data from the dynamic and static scans, whereas the maximum standardized uptake value (SUV_max_) and the tumor-to-blood standard uptake ratio (SUR) were derived from the static scans. These results were compared with pathology findings to evaluate diagnostic performance.

## Methods

### Study subjects

This prospective clinical study enrolled patients with indeterminate pulmonary lesions identified on CT scan and a clinical indicator for [^18^F]FDG PET/CT at the General Hospital of Ningxia Medical University between October 2019 and June 2021. Clinical indications included suspicion of malignancy based on CT findings, such as lesion size, irregular borders, or growth on follow-up imaging, or clinical symptoms suggestive of lung cancer. This study was approved by the ethics committee of the General Hospital of Ningxia Medical University (Approval No. 2019 − 529) and was registered in the Chinese Clinical Trial Registry (ChiCTR, ChiCTR2400081858. Registered 14 March 2024 – Retrospectively registered, https://www.chictr.org.cn/showproj.html?proj=188204). Informed consent was obtained from all participants. Following PET/CT imaging, a 3-year follow-up period was implemented to confirm definitive pathological diagnoses, which were obtained via CT-guided percutaneous lung biopsy or surgical excision. Patients without pathological validation were excluded from further analysis.

### [^18^F]FDG PET/CT protocol

All patients were instructed to fast for at least 6 h before imaging with blood glucose levels confirmed to be below 10 mmol/L. Weight, height, and blood glucose levels were recorded before intravenous [^18^F]FDG administration.

All [^18^F]FDG PET/CT imaging was performed using a Discovery VCT PET/CT scanner (GE HealthCare, Waukesha, WI, USA). During each scan, low-dose CT (120 kV, according to the weight) was performed for subsequent attenuation correction. Dynamic PET acquisition over the thorax started simultaneously with [^18^F]FDG injection (335.37 ± 145.12 MBq, *n* = 40), consisting of 33 frames: 10 × 3 s, 9 × 10 s, 8 × 15 s, and 6 × 60 s. Standard static PET/CT scanning (3-dimensional acquisition; 3 min per bed position and seven bed positions per patient) from skull to mid-thigh was performed approximately 50 min post-injection, followed by delayed static imaging (one or two bed positions, 3 min per bed position) covering the pulmonary lesions at approximately 2.5 h post-injection. PET data were reconstructed using an iterative algorithm into a 128 × 128 matrix and slice thickness of 3.27 mm.

### PET data analysis

PET data evaluation was conducted using Carimas version 2.10 [10]. Three-dimensional (3D) volumes of interest (VOIs) around the pulmonary lesions were carefully delineated by experienced nuclear medicine physicians on fused PET/CT images acquired across the dynamic, standard static, and delayed static phases. Consistent VOI placement was ensured by using anatomical landmarks from the accompanying low-dose CT. The mean activity concentration (Bq/mL) of the lesion VOI in the static scans was appended to the dynamic TAC at the mid-point of the static windows. A comprehensive lesion TAC extending up to 2.5 h post-injection was generated by combining the data from the 10-min dynamic acquisition and the static uptake values derived from the same lesion VOI in the standard static (50 min) and delay static (2.5 h) scans. The radioactive decay was corrected relative to the injection time at all time points.

Image-derived input functions (IDIFs) were obtained from a fixed-size cylindrical VOI (approximately 9000 mm^3^) placed within the descending thoracic aorta. The VOI position, orientation, and shape were manually refined on a per-slice basis to minimize spill-in contamination and ensure accurate containment within the aortic lumen, while consistent dimensions were maintained across all patients. A 2.5-h IDIF curve was generated by combining the dynamic aorta TAC (first 10 min) with the activity concentrations from the aorta VOI in the standard static and delayed static scans using a similar method as that used for the lesion TAC.

The [^18^F]FDG kinetics were analyzed using different compartment models with a 2.5-h IDIF as the input function. For Patlak analysis, uptake values from the VOI at 10 min, 50 min, and 2.5 h were used to derive the net influx rate (K_i_) and intercept. Linearity of the Patlak plot using the three time points was visually confirmed for every region of interest (ROI). The 2.5-h TAC of the VOI was analyzed using an irreversible two-tissue compartment model (2TCM irreversible) with three rate constants (K_1_, k_2_, k_3_) where K_i_ was calculated as K_i_ = K_1_k_3_/(k_2_ + k_3_). The same TAC was also analyzed using a reversible 2TCM model with four rate constants (K_1_, k_2_, k_3_, k_4_), where K_i_ was calculated as K_i_ = K_1_k_3_/(k_2_ + k_3_ + k_4_). All kinetic parameters (K_1_, k_2_, k_3_, k_4_, and K_i_) were estimated by fitting the compartment models using Carimas software.

For the semi-quantitative analysis, the SUV_max_ and SUR were calculated from the static PET acquisitions. [^18^F]FDG uptake was assessed by delineating a 3D ROI around the pulmonary lesions, and the SUV_max_ was measured. The SUR was defined as the SUV_max_ of the lesion divided by the SUV_max_ of the ROI centered in the descending thoracic aorta: SUR = SUV_max_ (Lesion)/SUV_max_ (Aorta).

### Statistical analyses

Statistical analyses were conducted using OriginPro 2018 software (Origin Lab Corporation, Northampton, MA, USA). Data normality was assessed using the Shapiro–Wilk normality test. Normally distributed data are presented as the mean ± standard deviation and were analyzed with independent samples analysis of variance (ANOVA) to compare the PET parameter (delayed SUR) between the malignant and non-malignant groups. Non-parametric distributed data were presented as the median [range] and analyzed with non-parametric Kruskal–Wallis ANOVA for comparisons between groups (K_1_, k_2_, k_3_, k_4_, K_i_, intercept, SUV_max_, delayed SUV_max_, and SUR). Statistical significance was set at *P* < 0.05. To evaluate the diagnostic accuracy of each dynamic and static PET parameter, receiver operating characteristic (ROC) curve analysis was performed. Area under the curve (AUC) values, optimal cut-off points (determined by maximizing Youden’s index), sensitivity, and specificity were calculated to assess the ability of these parameters to differentiate between malignant and non-malignant lesions.

## Results

### Patients

**Fig. 1:**
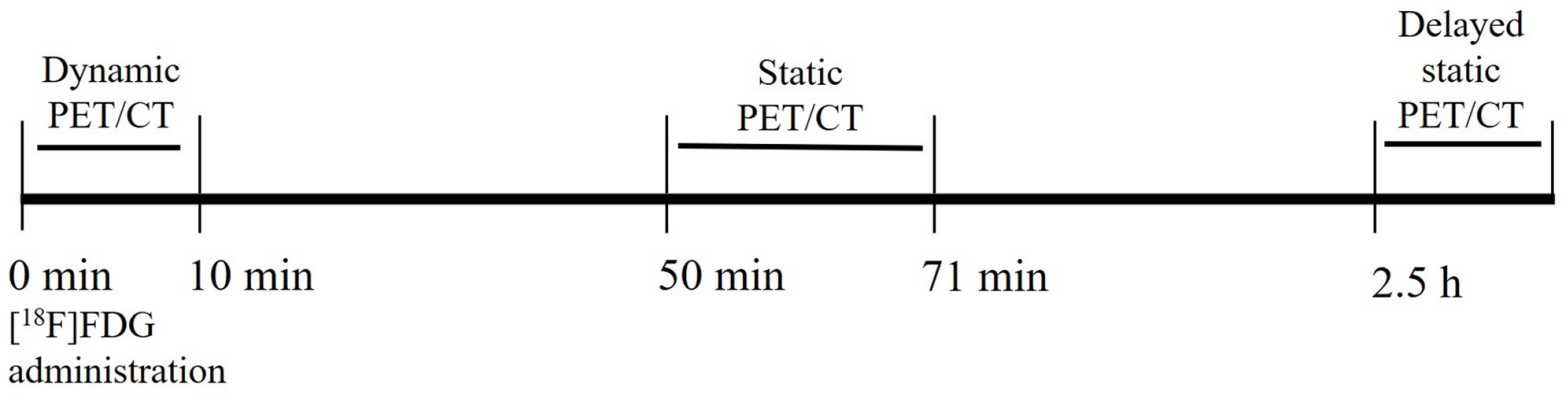
Protocol for [¹⁸F]FDG PET/CT

**Fig. 2:**
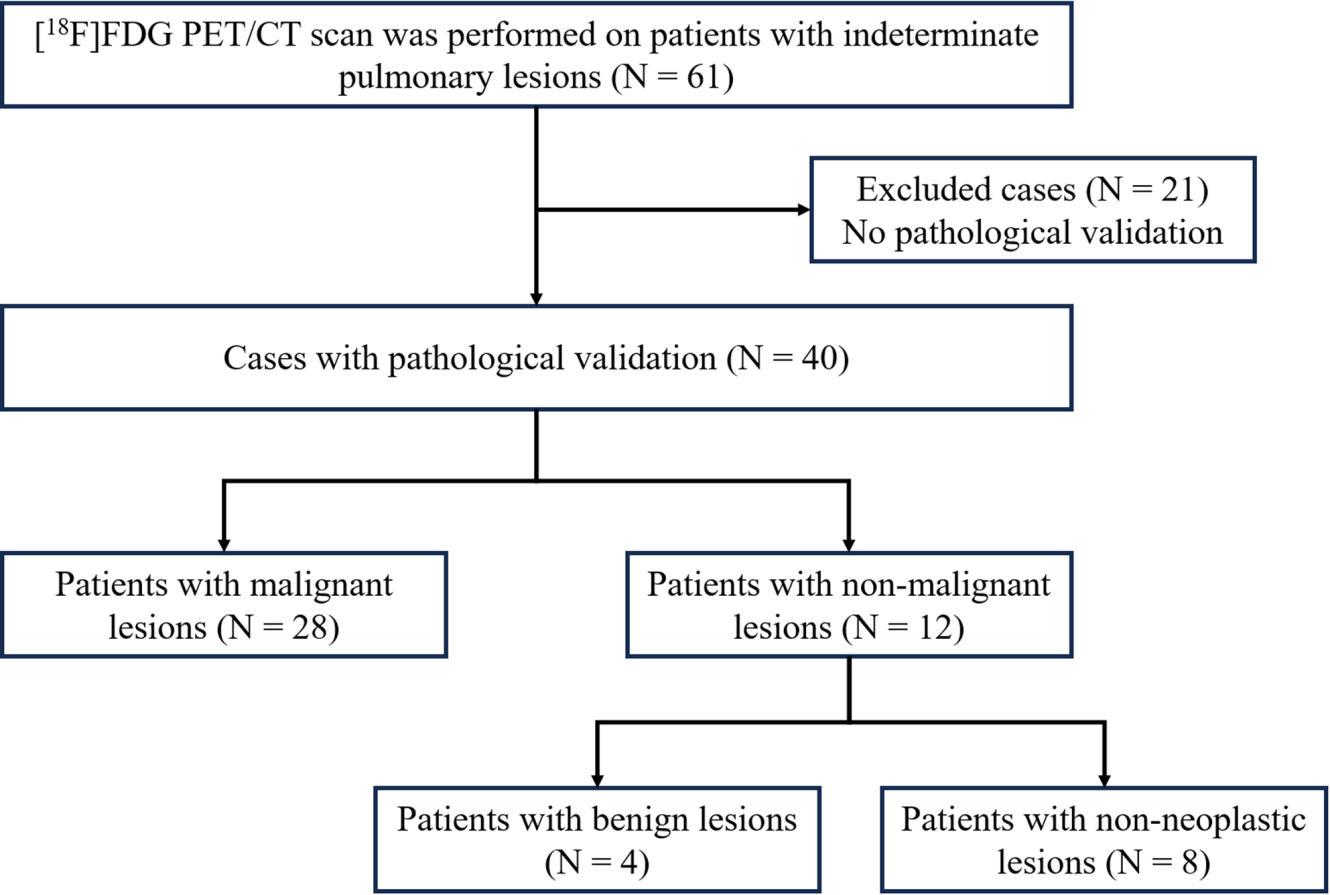
Flow chart for patient enrollment

A total of 61 patients with indeterminate pulmonary lesions were initially enrolled in this study. All patients underwent [^18^F]FDG PET/CT protocol (Fig. 1), which included a 10-min dynamic PET scan initiated simultaneously with the [^18^F]FDG injection. A standard static whole-body scan commenced at an average of 51 ± 6 min post-injection (*n* = 61), and a delayed static scan focused on the thorax started at 166 ± 30 min post-injection. After a 3-year follow-up period, 40 patients received a definitive pathological diagnosis, and 21 were excluded due to the absence of pathological confirmation. Of the 40 patients with confirmed diagnoses, 28 were diagnosed with malignant pulmonary lesions. The lesions of the remaining 12 patients were classified as non-malignant (4 benign lesions and 8 non-neoplastic lesions). A flow chart of patient enrollment is shown in Fig. 2, and Table 1 presents a detailed summary of patient characteristics.

**Table 1:** Patient characteristics and administered doses of [^18^F]FDG

Category	Total	Patients with malignant lesions	Patients with non-malignant lesions
Sex (male/female)	24/16	17/11	7/5
Age (years)	63 ± 8	63 ± 7	63 ± 10
Height (cm)	167.1 ± 7.5	168.3 ± 6.7	164.4 ± 8.8
Weight (kg)	66.45 ± 11.71	64.8 ± 11.7	71.1 ± 11.7
[^18^F]FDG dose (MBq)	335.37 ± 145.12	327.18 ± 139.22	353.81 ± 162.49

Among the 28 malignant cases, histological examination identified 21 adenocarcinoma cases, 4 squamous cell carcinoma cases, 2 small cell lung cancer cases, and 1 large cell neuroendocrine carcinoma case. Lymph node involvement was infrequent, with only two cases demonstrating nodal metastases. Nineteen patients underwent molecular pathology. Of these, epidermal growth factor receptor (EGFR) mutation status was assessed in 13 patients, with 12 (92.3%) testing positive. Anaplastic lymphoma kinase (ALK) rearrangement testing was conducted in 12 patients, all of whom had negative results. Programmed death-ligand 1 (PD-L1) expression was evaluated in five patients, with no positive results. The 12 non-malignant cases included 7 instances of tuberculosis and granulomatous inflammation, 4 cases of organizing pneumonia and fibrosis, and a case of inflammatory pseudotumor. A detailed summary of the histopathological characteristics of the pulmonary lesions is presented in Table 2.

**Table 2:** Histopathological characteristics of pulmonary lesions

Characteristics	Distribution
Histopathological type of malignant pulmonary lesion (N)	28
Adenocarcinoma	21
Squamous cell carcinoma	4
Small cell lung cancer	2
Large cell neuroendocrine carcinoma	1
Lymph node metastases	2/28
Molecular pathology of malignant pulmonary lesion (N)	19
EGFR mutation	12/13
ALK rearrangement	0/12
PD-L1 expression	0/5
Histopathological type of non-malignant pulmonary lesion (N)	12
Tuberculosis and granulomatous inflammation	7
Organizing pneumonia and fibrosis	4
Inflammatory pseudotumor	1

### Comparison of PET parameters

**Fig. 3:**
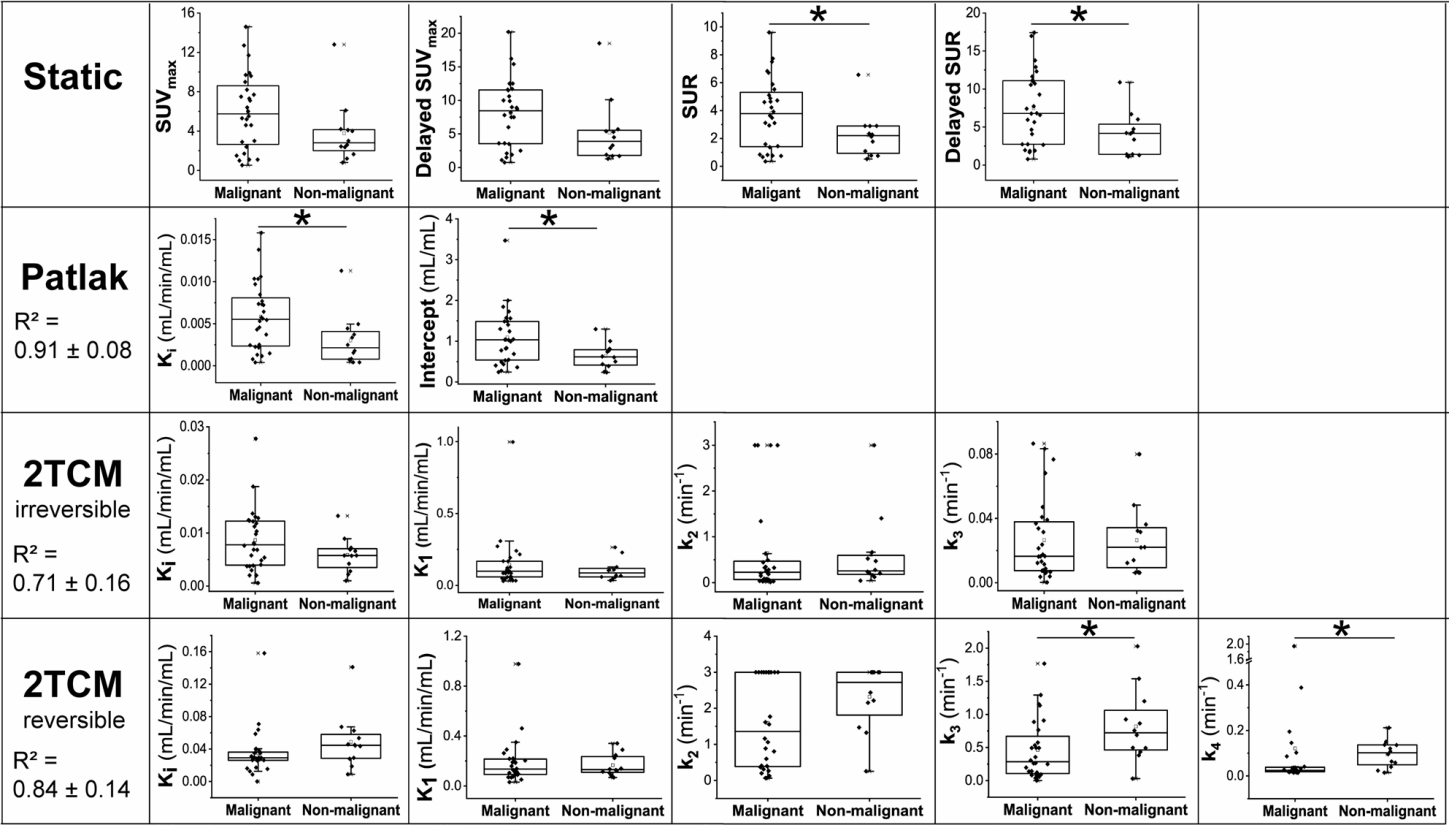
Comparison of semi-quantitative and kinetic parameters between patients with malignant and non-malignant lesions. SUV_max_ and SUR were derived from static analysis, while kinetic parameters were estimated using Patlak graphical analysis and reversible and irreversible two-tissue compartment models (2TCM). *p < 0.05

Table 3; Fig. 3 illustrate the quantitative and semi-quantitative PET parameters (K_i_, K_1_, k_2_, k_3_, k_4_, intercept, SUV_max_, delayed SUV_max_, SUR, and delayed SUR) for malignant and non-malignant lesions. Kinetic parameters (K_1_, k_2_, k_3_, k_4_, and K_i_) were derived from the time-activity curves generated by combining the 10-min dynamic data with the standard and delayed data, as described in the Methods section. All Patlak and 2TCM fits converged successfully without exclusion or manual intervention. Low R^2^ values observed in a minority of cases were retained in subsequent analyses to avoid selection bias and preserve the full dataset.

**Table 3:** Kinetic parameters derived from static analysis, Patlak graphical analysis, and reversible and irreversible two-tissue compartment models (2TCM)

	Parameter	Malignant	Non-malignant	Median Difference (95% CI)	*P*-value
**Static**	SUV_max_	5.75 [0.53–14.60]	2.80 [0.80–12.80]	2.30 [-0.10, 4.87]	0.074
	Delayed SUV_max_	8.45 [0.75–20.20]	3.90 [1.30–18.50]	3.35 [-0.20, 6.70]	0.063
	SUR	3.78 [0.35–9.61]	2.20 [0.52–6.56]	1.70 [0.03, 3.05]	0.045
	Delayed SUR	6.79 [0.78–17.42]	4.18 [1.10–10.88]	2.83 [0.39, 6.32]	0.029
**Patlak**	K_i_ (mL/min/mL)	0.00554 [0.00041–0.01581]	0.00214 [0.00041–0.01130]	0.00277 [0.00046, 0.00525]	0.023
	Intercept (mL/mL)	1.03385 [0.24368–3.47164]	0.61592 [0.23596–1.29731]	0.38645 [0.03876, 0.75631]	0.021
**2TCM irreversible**	K_i_ (mL/min/mL)	0.00778 [0.00059–0.02778]	0.00576 [0.00101–0.01323]	0.00221 [-0.00085, 0.00560]	0.184
	K_1_ (mL/min/mL)	0.09773 [0.02992–0.99748]	0.08674 [0.03415–0.26436]	0.00708 [-0.02558, 0.05173]	0.813
	k_2_ (/min)	0.25502 [0.01124–3.00000]	0.25645 [0.04267–3.00000]	-0.08081 [-0.24001, 0.12164]	0.376
	k_3_ (/min)	0.01639 [0.00020–0.08625]	0.02201 [0.00622–0.07990]	-0.00085 [-0.01482, 0.01008]	0.791
**2TCM reversible**	K_i_ (mL/min/mL)	0.02910 [0.00000–0.15806]	0.04463 [0.00881–0.14093]	-0.01504 [-0.02736, 0.00030]	0.059
	K_1_ (mL/min/mL)	0.13576 [0.03119–0.97722]	0.13115 [0.06794–0.34035]	-0.00669 [-0.05403, 0.06507]	0.768
	k_2_ (/min)	1.35760 [0.06385–3.00000]	2.71834 [0.25361–3.00000]	-0.66239 [-1.83942, 0.00000]	0.160
	k_3_ (/min)	0.28552 [0.00000–1.76664]	0.72208 [0.03017–2.02786]	-0.35179 [-0.65991, -0.00294]	0.039
	k_4_ (/min)	0.02477 [0.01367–1.94431]	0.10196 [0.01453–0.21183]	-0.04481 [-0.09625, -0.00664]	0.018

In static analysis, neither the SUV_max_ nor the delayed SUV_max_ was distinguishable between malignant and non-malignant tissues (5.75 [0.53–14.6] vs. 2.80 [0.80–12.80] and 8.45 [0.75–20.20] vs. 3.90 [1.30–18.50], respectively). However, SUR derived from both the standard and delayed static scans demonstrated statistically significant differences between the groups (*P* = 0.045, 3.78 [0.35–9.61] vs. 2.20 [0.52–6.56] and *P* = 0.029, 6.79 [0.78–17.42] vs. 4.18 [1.10–10.88], respectively).

In dynamic analysis, Patlak graphical analysis identified a significant difference in K_i_ and intercept between malignant and non-malignant groups (*P* = 0.023, 0.00554 [0.00041–0.01581] mL/min/mL vs. 0.00214 [0.00041–0.01130] mL/min/mL and *P* = 0.021, 1.03385 [0.24368–3.47164] mL/mL vs. 0.61592 [0.23596–1.29731] mL/mL).

The irreversible 2TCM analysis showed no significant difference in K_i_ (0.00778 [0.00059–0.02778] mL/min/mL vs. 0.00576 [0.00101–0.01323] mL/min/mL), K_1_ (0.09773 [0.02992–0.99748] mL/min/mL vs. 0.08674 [0.03415–0.26436] mL/min/mL), k_2_ (0.25502 [0.01124–3.00000] min^-1^ vs. 0.25645 [0.04267–3.00000] min^-1^), or k_3_ (0.01639 [0.00020–0.08652] min^-1^ vs. 0.02201 [0.00622–0.07990] min^-1^) (all *P* > 0.05).

The reversible 2TCM analysis showed that both k_3_ and k_4_ were significantly lower in the malignant group compared to the non-malignant group (k_3_: *P* = 0.039, 0.28552 [0.00000–1.76664] min^-1^ vs. 0.72208 [0.03017–2.02786] min^-1^ ; k_4_: *P* = 0.018, 0.02477 [0.01367–1.94431] min^-1^ vs. 0.10196 [0.01453–0.21183] min^-1^). No significant differences were observed for K_i_ (0.02910 [0.00000–0.15806] mL/min/mL vs. 0.04463 [0.00881–0.14093] mL/min/mL), K_1_ (0.13576 [0.03119–0.97722] min^-1^ vs. 0.13115 [0.06794–0.34035] min^-1^), or k_2_ (1.35760 [0.06385–3.00000] min^-1^ vs. 2.71834 [0.25361–3.00000] min^-1^) (all *P* > 0.05).

### Diagnostic performance

**Fig. 4:**
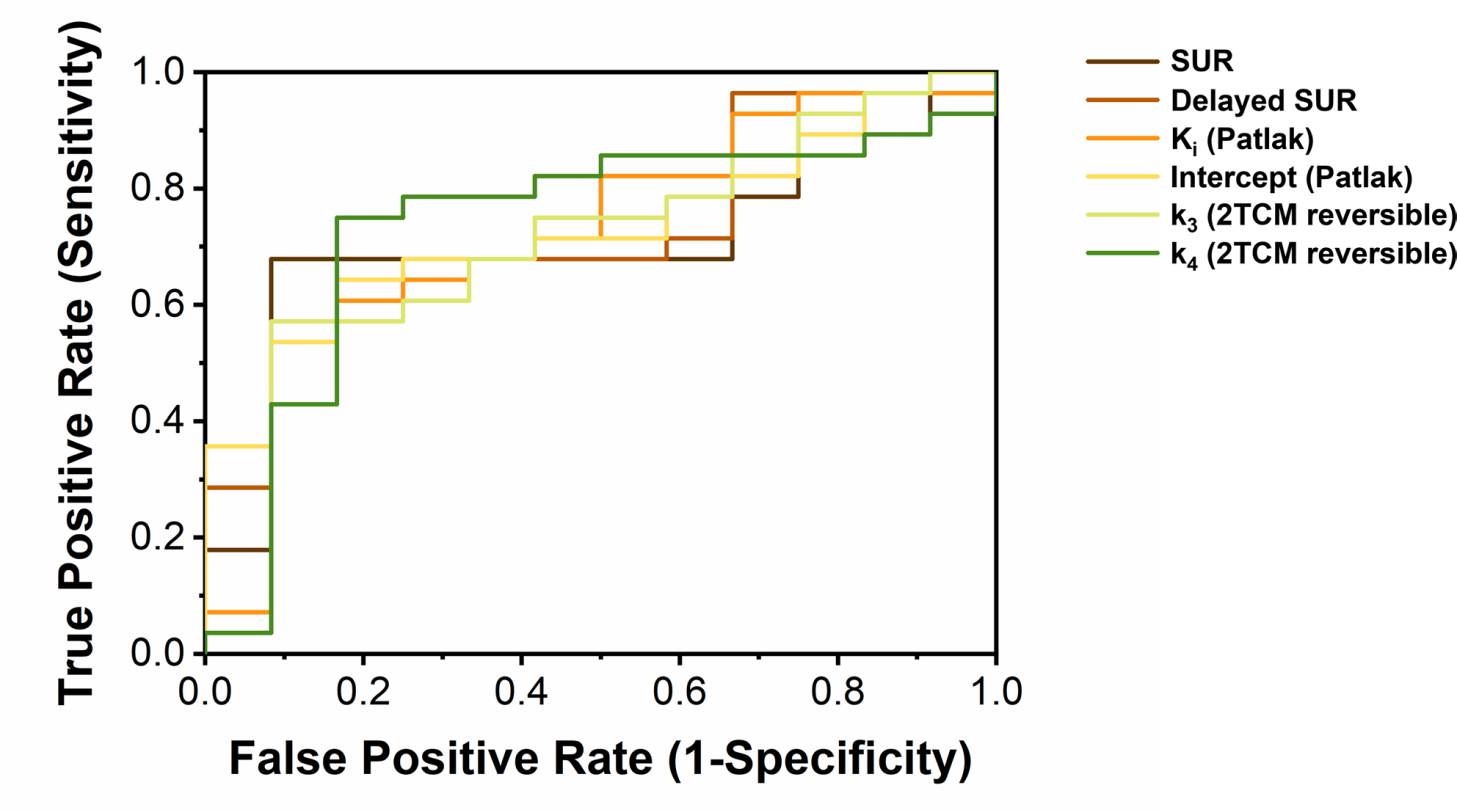
ROC curves comparing the diagnostic performance of various PET-derived metrics for differentiating malignant and non-malignant pulmonary lesions. The analysis includes semi-quantitative ratios (SUR and delayed SUR), Patlak graphical parameters (influx rate K_i_ and intercept), and kinetic rate constants (k_3_ and k_4_) derived from the reversible two-tissue compartment model (2TCM)

Following the selection of the Patlak and reversible 2TCM as the performing method for dynamic data analysis, ROC curve analysis was conducted to evaluate the diagnostic performance of parameters that showed significant group differences, including dynamic and static data (Fig. 4). Significant diagnostic accuracy for differentiating malignant from non-malignant lesions was demonstrated for SUR (AUC = 0.702 [95% confidence interval (CI): 0.491–0.914], *P* = 0.045), delayed SUR (AUC = 0.720 [95% CI: 0.531–0.910], *P* = 0.029), K_i_ (AUC = 0.729 [95% CI: 0.549–0.909], *P* = 0.023), intercept (AUC = 0.732 [95% CI: 0.540–0.925], *P* = 0.021), k_3_ (AUC = 0.708 [95% CI: 0.522–0.895], *P* = 0.039), and k_4_ (AUC = 0.738 [95% CI: 0.547–0.929], *P* = 0.018). The optimal cut-off values for these parameters, determined using Youden’s index, are presented in Table 4.

**Table 4:** ROC curve analysis and diagnostic performance

Parameter	Sensitivity	Specificity	Cut-off value	AUC [95% CI]	*P*-value
SUR	0.679	0.917	2.914	0.702 [0.491–0.914]	0.045
Delayed SUR	0.536	0.917	6.727	0.720 [0.531–0.910]	0.029
K_i_ (Patlak)	0.571	0.917	0.00514	0.729 [0.549–0.909]	0.023
Intercept (Patlak)	0.643	0.833	0.818	0.732 [0.540–0.925]	0.021
k_3_ (reversible 2TCM)	0.571	0.917	0.369	0.708 [0.522–0.895]	0.039
k_4_ (reversible 2TCM)	0.750	0.833	0.038	0.738 [0.547–0.929]	0.018

**Fig. 5:**
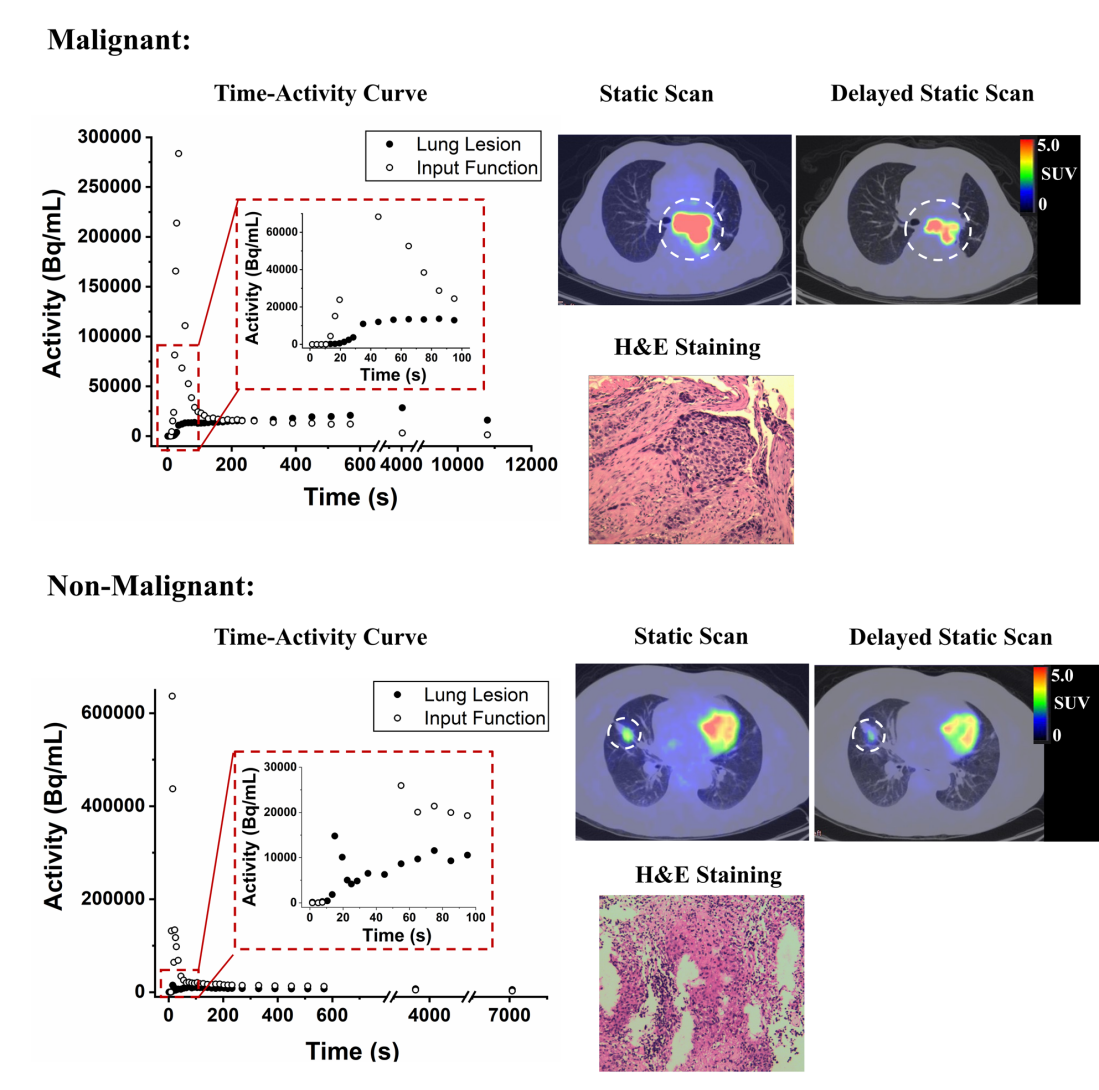
Representative time-activity curves derived from the descending thoracic aorta and lung lesion, PET/CT imaging of static and delayed static scan, and H&E staining of malignant and non-malignant lung tissue samples

A representative case of a malignant pulmonary lesion in a 62-year-old male patient is shown in Fig. 5. The [^18^F]FDG PET/CT scan revealed findings consistent with left lower lobe hilar-central lung cancer. Patlak kinetic analysis of the tumor lesion yielded a K_i_ value of 0.01380 mL/min/mL, intercept 3.47164 mL/mL. In the early static scan, SUV_max_ was 14.6 and SUR was 9.61. In the delayed static scan, SUV_max_ and SUR increased to 20.20 and 16.97, respectively. Bronchoscopic biopsy of the tracheal mucosa from the lower segment of the left main bronchus confirmed squamous cell carcinoma. Histopathological examination of hematoxylin and eosin (H&E) staining revealed intercellular bridges and evidence of keratinization, which are hallmark features of squamous cell carcinoma. Mitotic figures were also identified, indicating active proliferation within the tumor as the tumor invaded the surrounding fibrous stroma. Immunohistochemistry analysis revealed EGFR mutation positivity, whereas ALK rearrangement and PD-L1 expression were negative.

A typical non-malignant example from a 51-year-old female patient is also presented (Fig. 5). The [^18^F]FDG PET/CT scan revealed positive uptake in the right middle lobe, with the lesion measuring 2.2 × 2.5 cm. The Patlak kinetic analysis showed a K_i_ value of 0.00445 mL/min/mL, intercept 0.62545 mL/mL. In the early static scan, SUV_max_ and SUR was 4.20 and 2.90, respectively. In contrast, in the delayed static scan, SUV_max_ was 5.40 and SUR was 4.22. Bronchoscopic biopsy, stained with H&E, revealed characteristics consistent with granulomatous inflammation, characterized by a dense inflammatory infiltrate with numerous coalescing, non-necrotizing granulomas composed of epithelioid histiocytes with abundant pink cytoplasm and surrounded by lymphocytes. This granulomatous inflammation expanded interstitial spaces, effacing normal alveolar structures.

## Discussion

Incidental pulmonary nodules are observed in an increasing proportion of the population, and practical approaches for the classification and differentiation of malignant and non-malignant lesions are needed for early intervention and disease follow-up. PET imaging is a sensitive medical technique for the diagnosis of oncological and inflammatory diseases. [^18^F]FDG is widely available, making it amenable to a large patient base for routine clinical diagnosis. However, an [^18^F]FDG PET imaging methodology study is essential to optimize the use of [^18^F]FDG with increased diagnostic accuracy. This is particularly important for hospitals and regions where [^18^F]FDG is in practice the only available PET imaging agent.

This study evaluated the diagnostic performance of a clinically efficient [^18^F]FDG PET/CT protocol that integrates a short 10-min dynamic acquisition with subsequent two-phase static imaging for differentiating malignant from non-malignant pulmonary lesions. Our findings demonstrated that the net influx rate K_i_ derived from this time-efficient workflow, along with the tumor-to-blood uptake ratio SUR obtained from both standard and delayed static acquisitions, provided statistically significant diagnostic accuracy, outperforming SUV_max_ values. Based on our experience, this workflow may integrate into existing clinical protocols in centers where the 10-minute dynamic acquisition can be scheduled for the first patient daily and delayed imaging is already routine. Importantly, this approach achieves an optimal balance between diagnostic accuracy, patient comfort, and operational efficiency.

For the kinetic analysis, we evaluated three complementary approaches on the same extended TACs: Patlak graphical analysis, irreversible 2TCM (k_4_ = 0), and reversible 2TCM (k_4_ ≠ 0). Patlak graphical analysis, a robust graphical method for estimating the net influx rate K_i_ from multiple time acquisitions under irreversible trapping assumptions [11–14], proved most suitable for our abbreviated protocol. The method requires that quasi-equilibrium between the reversible and irreversible compartments be established prior to the onset of the linear phase (t*). Consistent with this, incorporating the full early dynamic dataset (0–10 min) markedly degraded fit quality. Accordingly, only the final frame of the 10-min dynamic acquisition together with the static acquisitions at 50 min and 2.5 h were retained for Patlak analysis. While sparse late-time sampling may theoretically introduce minor bias or increased variance from un-sampled subtle non-linearities or noise sensitivity, the resulting K_i_ values remain in close agreement with those obtained from continuous late dynamic protocols with reported low biases [15]. The Patlak plot requires only two parameters K_i_ and intercept, and is inherently robust to noise in the early frames and to modest violations of the irreversible-trapping assumption once equilibration has occurred. The late static points we already acquire for clinical staging therefore provide exactly the data Patlak needs, yielding stable, clinically relevant K_i_ estimates that were the only kinetic parameter to show a statistically significant difference between malignant and non-malignant lesions. Although the Patlak intercept, which represents the apparent distribution volume of non-metabolized [¹⁸F]FDG, was significantly higher in malignant lesions, this finding should be interpreted with caution, as the intercept is susceptible to confounding influences such as tumor heterogeneity and IDIF-related biases including partial volume effects. It should therefore not be regarded as a reliable diagnostic metric [16].

Although K_i_ derived from irreversible 2TCM can offer superior diagnostic performance compared with SUV_max_ across various tumor types [17–19], in our study, the irreversible 2TCM (3 parameters) produced poorer fits (median R^2^ = 0.71 ± 0.16) and failed to detect any significant group differences in K_i_, K_1_, k_2_, or k_3_. The short 10-min dynamic window supplies limited information about the phosphorylation step (k_3_), and the forced k_4_ = 0 assumption is probably inappropriate for the inflammatory non-malignant lesions in our cohort, where some dephosphorylation occurs. These factors lead to biased or poorly identifiable micro-parameters and a K_i_ that does not discriminate the groups.

In contrast, the reversible 2TCM yielded a clearly superior fit (R^2^ = 0.84 ± 0.14), confirming the presence of measurable reversibility (k_4_ > 0) in our data. However, despite this improved fit, the reversible 2TCM produced unstable K_i_ estimates (calculated as K_1_k_3_/(k_2_ + k_3_ + k_4_)). Further examination of the individual rate constants revealed that both k_3_ and k_4_ were significantly lower in malignant lesions, each showing a negative correlation with malignancy. The reduction in k_4_ among malignant lesions is consistent with greater irreversible [^18^F]FDG trapping in tumor tissue, a finding that contrasts with the elevated k_4_ observed in inflammatory non-malignant lesions, where tracer washout is more prominent. The interpretation of the lower k_3_ in malignancies is less straightforward. One plausible explanation is that the 10-minute dynamic acquisition window may be insufficient to reliably resolve the individual kinetic steps of glucose transport and phosphorylation, thereby limiting the identifiability of k_3_ as a distinct parameter. It should be noted that R^2^ reflects goodness-of-fit but does not substitute for parameter uncertainty metrics; thus, the precision and identifiability of these micro-parameters cannot be fully assessed within the constraints of Carimas.

In previous studies, accurate estimation of K_i_ typically requires dynamic acquisitions of 30 min or longer [20], which presents practical challenges in routine clinical settings. Furthermore, without a total-body scanner, these dynamic scans cannot provide whole-body PET imaging, which is crucial for detecting distant metastases. Our protocol specifically aimed to tackle this challenge by using a significantly shorter 10-min dynamic scan combined with data from two static acquisitions to construct extended time-activity curves for both the lesion and IDIF. The K_i_ value derived this workflow significantly differentiated malignant from non-malignant pulmonary lesions, supporting the feasibility of obtaining valuable kinetic information from an abbreviated dynamic acquisition when complemented by later static time points. Our methodology aligns with the principle demonstrated by Samimi et al. of integrating a short dynamic acquisition with a later static scan to enable comprehensive kinetic modeling, with their study relying on simulated data for protocol evaluation [21].

In addition to kinetic analysis, our results highlight the practical value of the semi-quantitative SUR. The improved performance of SUR compared with SUV_max_ is likely attributable to its normalization to blood pool activity, which can reduce variability due to differences in tracer delivery or plasma clearance across subjects [22]. Furthermore, the significant performance of delayed SUR suggests an improvement in diagnostic value with delayed imaging. This enhanced accuracy of delayed SUR is potentially due to facilitated tracer washout from normal tissues, which enhances the contrast between malignant and non-malignant lesions. This observation is consistent with that of previous studies demonstrating the benefits of delayed imaging [23]. Clinically, therefore, the ability of SUR and delayed SUR to match the diagnostic performance of Patlak-derived K_i_ in the current cohort underscores the practical value of this protocol even for centers relying primarily on static PET acquisitions. The current study demonstrates that by adopting SUR instead of SUV_max_ as the primary semi-quantitative metric, clinicians can meaningfully improve the diagnostic accuracy of standard and delayed static PET/CT scans for pulmonary lesion characterization, without additional scanner time or specialized software beyond what is required for routine PET/CT reading.

This study has some limitations. First, the 10-min dynamic window plus two late static points provides far fewer data points than a conventional 30–60 min dynamic acquisition. Although Patlak analysis mitigated this limitation, full compartmental models remain sensitive to the reduced early-phase information. Regarding the delayed static scan, although the protocol specified a 2-hour acquisition, practical scheduling that patients frequently arrived beyond this target, resulting in a mean actual acquisition time of approximately 2.5 h. This timing remains well within the 90–270 min window reported to be effective for dual-time-point imaging and still allowed significant diagnostic improvement with delayed SUR compared with standard SUV_max_ [24]. Second, the use of IDIFs from the descending thoracic aorta introduces potential sources of bias, including partial-volume effects, respiratory motion, and spill-in from surrounding tissues. Due to software limitations and the focus on clinical feasibility, no formal partial-volume correction, motion correction, or delay/dispersion fitting was applied. These factors may affect the absolute accuracy of kinetic parameter estimates, particularly in patients with respiratory motion artefacts. Future studies should consider applying motion correction and partial-volume effect corrections to improve IDIF accuracy in thoracic imaging. Third, manual VOI delineation introduces operator-dependent variability that may affect reproducibility in broader clinical practice. Although VOIs were carefully delineated by experienced nuclear medicine physicians using fused PET/CT images and the same VOI boundaries were maintained consistently across dynamic, standard static, and delayed static phases for each patient, no formal inter-observer reliability assessment was performed. Semi-automatic or AI-assisted segmentation would improve reproducibility for the clinical application. Fourth, the relatively small sample size, especially within the benign and non-neoplastic subgroups, limits the generalizability of our findings. The modest AUC values (0.70–0.74) observed across Ki, SUR, and delayed SUR are comparable, and marginal differences between metrics should not be over-interpreted given the sample size constraints. As multiple parameters were examined without correction for multiple comparisons, the present findings should be regarded as exploratory and hypothesis-generating rather than confirmatory. Additionally, the absence of parameter uncertainty metrics, such as standard errors or coefficients of variation, limits the assessment of kinetic parameter identifiability and estimation robustness, a constraint inherent to the software employed. Validation in larger, prospectively designed, and better-balanced cohorts is warranted to confirm these findings.

## Conclusions

In conclusion, this study validates a clinically efficient [^18^F]FDG PET/CT protocol that integrates a 10-minute dynamic scan with two-phase static imaging. Our results show that the net influx rate K_i_ from Patlak graphical analysis, alongside the semi-quantitative tumor-to-blood uptake ratios SUR and delayed SUR, significantly differentiate malignant from non-malignant pulmonary lesions compared to conventional SUV_max_. Notably, SUR and delayed SUR can be computed from the routine static acquisitions already embedded in the protocol, without additional scan time or specialized kinetic software, making them immediately applicable in standard clinical practice. The dynamic component may integrate into existing workflows in appropriately equipped centers. Overall, these findings support the diagnostic utility of this time-efficient protocol for pulmonary lesion characterization. Notably, SUR and delayed SUR performed comparably to K_i_, suggesting that full kinetic modeling may not be necessary in all clinical scenarios and that semi-quantitative parameters alone may suffice in routine practice.

## Data Availability

The datasets used or analyzed during the current study are available from the corresponding author on reasonable request.

## Abbreviations

[^18^F]FDG 2: Deoxy-2-[^18^F]fluoroglucose
2TCM: Two-tissue compartment model
3D: Three-dimensional
ALK: Anaplastic lymphoma kinase
AUC: Area under the curve
CT: Computed tomography
EGFR: Epidermal growth factor receptor
IDIF: Image-derived input function
PD-L1: Programmed death-ligand 1
PET: Positron emission tomography
PET/CT: Positron emission tomography/computed tomography
ROC: Receiver operating characteristic
ROI: Region of interest
SUR: Standard uptake ratio
SUVmax: Maximum standardized uptake value
TAC: Time-activity curve
VOI: Volume of interest
